# Effects of duration of a plant-based diet stimulus at first feeding on nutritional programming in Atlantic salmon (*Salmo salar*)

**DOI:** 10.1007/s10695-026-01639-7

**Published:** 2026-02-17

**Authors:** Xu Gong, Matthew Sprague, Stuart McMillan, Pedro Gómez-Requeni, Fernando Norambuena, Sam A. M. Martin, Douglas R. Tocher, Mónica B. Betancor

**Affiliations:** 1 Institute of Aquaculture, Faculty of Natural Sciences, University of Stirling, Stirling, FK9 4LA Scotland, UK; 2 BioMar A/S, Mylius Erichsensvej 35, 7330 Brande, Denmark; 3 BioMar A/S, Havnegata 9, Pirsenteret 3, 7010 Trondheim, Norway; 4 Fish Immunology Research Centre, University of Aberdeen, Aberdeen, AB24 2TZ Scotland, UK; 5 Guangdong Provincial Key Laboratory of Marine Biotechnology, Shantou University, Shantou , Guangdong, 515063 China

**Keywords:** Atlantic salmon, Nutritional challenge, Fatty acid, LC-PUFA biosynthesis, Gene expression

## Abstract

Nutritional programming could potentially be a strategy for sustainable aquaculture to adapt fish to feeds formulated with low levels of marine ingredients. The present study examined the effects of early dietary stimulus duration and the impacts of a later challenge with a similar diet. At first feeding, Atlantic salmon (*Salmo salar*) fry of ~ 0.15 g initial weight were fed a vegetable diet (77% plant-based ingredients) for either 1 (V1) or 2 weeks (V2), while a control group (M) received a marine diet (86% marine-based ingredients) for the 2 weeks (stimulus phase). All fish then received a marine diet for 16 weeks (intermediate phase) before being refed a vegetable diet for 6 weeks (challenge phase). Water temperature was 13 °C during the trial, and the only difference between the three experimental groups (V1, V2 and M) was their dietary regime during the first 2 weeks. Obviously, stimulus duration had no phenotypic impacts on growth or tissue fatty acid compositions of either total or polar lipids after challenge. However, the expressions of key genes of LC-PUFA biosynthesis were upregulated significantly in the V1 group, whereas the expressions of most transcription factors were downregulated in the V2 group. After the challenge, the circular muscle thickness of the intestine in the V1 group was increased by 15%, and goblet cell numbers were reduced by 15%. Overall, further investigations about the duration of the stimulus and assessment with longer challenges are required to elicit positive effects and to refine the use of nutritional programming in Atlantic salmon.

**Supplementary Information:**

The online version contains supplementary material available at 10.1007/s10695-026-01639-7.

## Introduction

The ingredients used traditionally in aquafeeds, fish oil (FO) and fishmeal (FM), are finite marine resources (Hodar et al. 2020; Aas et al. 2022), which are being replaced substantially by a range of alternatives derived from algae, animal, insect, single-cell sources and, in particular, plant (crop) products. However, studies on FM and FO replacement in different teleost species have reported negative effects on fish growth and health performance (Turchini et al. 2011; Aas et al. 2022). The efficacy of the application of plant-based ingredients can be restricted by several factors, such as relative deficiencies in some essential amino acids, the presence of anti-nutritional factors, and digestibility issues related to carbohydrate content, especially in carnivorous fish species (Gatlin et al. 2007; Glencross et al. 2007). In addition, while vegetable oils (VO) can have a varied fatty acid profile, they are characteristically rich in oleic acid (18:1n-9), linoleic acid (LA, 18:2n-6) and/or α-linolenic acid (ALA, 18:3n-3), but lack the long-chain polyunsaturated fatty acids (LC-PUFA), arachidonic (ARA; 20:4n-6), eicosapentaenoic (EPA; 20:5n-3) and docosahexaenoic (DHA; 22:6n-3) acids (Turchini et al. 2011), that have essential functional roles in cell membrane structure, metabolic regulation, inflammatory and immune responses, and neural system development (Tocher 2003, 2010). Although salmonids like Atlantic salmon (*Salmo salar*) are able to biosynthesize LC-PUFA from the C_18_ precursors ALA and LA, additional EPA and DHA are required in the diet for optimum growth and health (Sprague et al. 2019; Lutfi et al. 2022). Despite major progress in the application of plant-based feeds for Atlantic salmon, the potential negative effects on growth, feed efficiency and health, as well as the deleterious impact on nutritional quality for human consumers in terms of reduced EPA and DHA levels in fish fillet, remain a challenge (Sprague et al. 2016).

Long-term adaptations in metabolism and physiology, stimulated by specific events and driven by mechanisms like epigenetic modifications, may occur in mammals during critical early developmental stages such as gestation and lactation (Gluckman et al. 2005; Lucas 1998; Patel and Srinivasan 2002). If the event is a dietary or nutrient-based stimulus, the phenomenon has been termed “nutritional programming.” For example, research in rodents indicated that a maternal low-protein diet during pregnancy led to reduced adipocyte cell size in offspring, which was associated with elevated lipolysis (Ferland-McCollough et al. 2012). In addition, offspring were more likely to suffer obesity or metabolic syndromes if females were over-nourished by a high-fat diet during pregnancy or lactation in rats (Kirk et al. 2009). Also, a maternal high saturated fatty acid diet reduced the biosynthesis of n-3 LC-PUFA in offspring when they were fed ALA and LA diets (Kelsall et al. 2012). The authors suggested that a high fat/high saturated fatty acid diet decreased the expression of the *FADS2* gene (Kelsall et al. 2012), which encodes delta-6-desaturase (∆6 FAD) enzyme, the rate-limiting enzyme of LC-PUFA biosynthesis (Cho et al. 1999), by epigenetic modifications.

Nutritional programming during early development can be applied to improve nutritional utilisation, health, and disease resistance that occur later in life (Rodríguez-González et al. 2020), and several studies have demonstrated that nutritional programming can confer beneficial effects on farmed fish. For example, after an acute nutritional stimulus with plant-based diets, rainbow trout (*Oncorhynchus mykiss*) fry showed better growth, feed intake, feed efficiency and carbohydrate digestion when challenged by a similar diet later in life (Geurden et al. 2007, 2013). Moreover, nutritional programming improved the utilisation of plant-based diets in gilthead sea bream (*Sparus aurata*), with partial replacement of FO by VO in the parental diet inducing long-term molecular impacts on n-3 LC-PUFA and energy metabolism in the liver of offspring (Izquierdo et al. 2015; Turkmen et al. 2017). The liver is the most active metabolic tissue in fish species with respect to lipid metabolism, including LC-PUFA synthesis, and changes in dietary fatty acid composition can induce important transcriptional responses in the genes encoding the endogenous biosynthesis of EPA and DHA from ALA (Betancor et al. 2015; Monroig et al. 2010).

In our previous study in Atlantic salmon, a diet with low n-3 LC-PUFA (EPA and DHA) was applied over a 3-week stimulus period from first feeding, and the potential of nutritional programming to increase nutrient utilisation efficiency was determined in older fish (15–20 g) challenged with a similar plant-based diet (Clarkson et al. 2017). Although the concept was clearly demonstrated, the stimulus of the 3-week vegetable diet also resulted in significantly lower growth than the marine group (control) at the end of the stimulus phase. Thus, the specific aims of the present study were (1) to determine if nutritional programming could be triggered by a shorter (1 or 2 weeks) stimulus period, (2) to assess whether nutritional responses were affected by the duration of the stimulus, and (3) to evaluate whether the capacity for the biosynthesis of EPA and DHA during the challenge phase was affected by the duration of the stimulus. Consequently, lipid profiles together with selected molecular markers and histological evaluation of different tissues at key points during the trial were determined to assess nutritional programming as a potential strategy to partially mitigate problems encountered with the replacement of marine ingredients in aquafeeds, reduce the impact of the limited global supply of n-3 LC-PUFA, and promote the sustainability of aquaculture.

## Materials and methods

### Ethics statement

Animals were handled in accordance with the Animals Scientific Procedures Act 1986 (Home Office Code of Practice, HMSO: London January 1997) and in accordance with European Directive 2010/63/EU. In addition, all procedures and protocols carried out in the present study were subjected to an ethics review by the University of Stirling Animal Welfare and Ethical Review Board and approved (AWERB No. 18 19/045/New ASPA) prior to the commencement of the study.

### Dietary trial

**Fig. 1:**
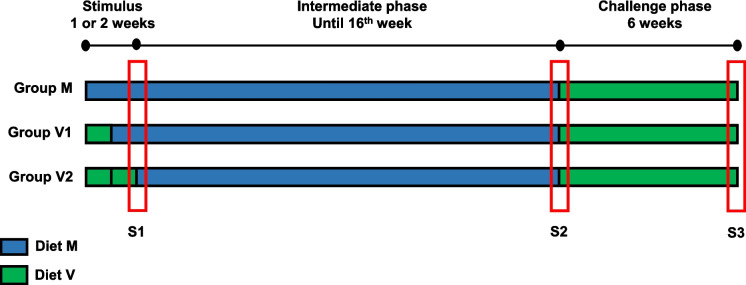
Design of the dietary feeding trial. S1 (sample point 1), end of stimulus; S2, end of the intermediate phase; S3, end of the challenge phase

Atlantic salmon eggs (388 degree-days [DD] post-fertilization) were obtained from Mowi (Scotland, UK) and transferred to the Niall Bromage Freshwater Research Unit (NBFRU) at the Institute of Aquaculture, University of Stirling. Eggs hatched after 500 DD with the fish remaining in the alevin stage for a further 350 DD. The incubation temperature was 7.0 ± 0.4 °C. At 850 DD, when salmon reached the fry stage with a 97.7% survival rate, 2700 fish were bulk-weighed in batches of 300 with 0.15 g average initial weight per fish and were distributed into 300 L tanks for the nutritional trial (nine tanks in total), which started at first feeding and continued for 24 weeks. Two types of experimental feeds were manufactured by BioMar A/S (TechCentre, Brande, Denmark) following formulations that included some mixed ingredients as is standard practice within commercial feed production (Table 1). The marine diets (M) contained FM and FO as the main protein and lipid sources, whereas the vegetable diets (V) were formulated to have high proportions of terrestrial plant protein sources and VO as the lipid source. The fatty acid compositions of the diets are shown in Table 1. The feeds were prepared at different pellet sizes (0.5 mm, 0.8 mm, 1.1 mm, 1.5 mm and 2.0 mm) and compositions (i.e. increasing lipid content) to be provided as the fish increased in size during the trial (Table 1). At first feeding, three tanks of fish (*n* = 3) were allocated to each of the three experimental groups termed V1, V2 and M as illustrated (Fig. 1). The V1 and V2 groups were fed the vegetable-based diet for either 2 week (V1) or 2 weeks (V2), respectively, while the third group (M) was fed the marine diet for both weeks. This initial 2-week period was termed as the “stimulus phase”. Thereafter, all fish were fed marine (M) diets until the end of week 16 “intermediate phase” at which point all groups were transferred back to a vegetable-based V diet for a further 6 week “challenge phase”. The feeds were provided to fish by automatic feeders (Arvo-tec TD2000, Huutokoski, Finland) combined with user interface (ArvoPRO) to ensure the acquisition of accurate data for feed provided to the tanks. Uneaten feed was collected twice a day after daily feeding using a syphon pipe with filter, before drying to enable accurate calculation of actual voluntary feed intake. The trial was carried out in tanks that were part of a single Recirculating Aquaculture System (RAS) at NBFRU. Water temperature was maintained at 13.1 ± 0.5 °C with a 24:0 light to dark regime. Oxygen level (86% saturation, 8–9 mg/L), pH (7.16 ± 0.2), nitrogen NO_2_ (0.32 ± 0.2 mg/L), total ammonia nitrogen (0.13 ± 0.7 mg/L) and chloride (135 ± 13 mg/L) were determined daily and controlled throughout the feeding trial.

**Table 1:** Ingredients (percentage) and analysed proximate and fatty acid compositions (percentage of total fatty acids) of the experimental feeds

	Stimulus phase		Intermediate phase			Challenge phase	
	0.5 mm	0.5 mm	0.8 mm	1.1 mm	1.5 mm	1.5 mm	2.0 mm
	**M**	**V**	**M**	**M**	**M**	**V**	**V**
*Feed ingredients*^*1*^* (%)*							
Fishmeal	66.7	–	53.0	53.0	49.3	5.0	5.0
Krill meal (56%)	10.0	2.5	7.0	7.0	–	–	–
Fish Peptones	5.0	2.5	5.0	5.0	–	5.0	5.0
Plant protein (SPC, wheat & maize gluten, pea protein)	3.5	70.6	16.4	16.4	26.1	60.0	60.0
Starch sources	6.5	4.0	5.5	5.5	9.5	8.1	8.1
Fish oil	4.4		9.8	9.8	8.6	–	–
Vegetable oil	–	6.7	0.5	0.5	5.1	14.1	14.1
Lecithin	0.5	3.6	0.6	0.6	0.5	0.5	0.5
Premixes (vits., minerals., AAs, nucl., antiox., yttrium)	3.0	10.9	3.0	3.0	2.5	8.8	8.8
*Proximate compositions*							
Dry matter (DM) (%)	94.5	94.5	93.4	91.5	96.9	92.9	95.2
Protein (% DM)	60.0	56.0	55.7	53.0	52.6	50.9	51.8
Lipid (% DM)	13.3	13.4	17.0	16.4	17.8	20.0	16.3
Ash (% DM)	12.2	8.8	11.1	10.0	10.1	7.3	7.3
Gross energy (KJ/g)	20.6	21.1	21.3	20.9	21.8	22.4	22.3
*FA composition (%)*							
**Σ SFA**^**2**^	29.0	17.0	28.6	28.7	20.9	10.1	10.4
**Σ MUFA**^**3**^	28.8	45.9	34.3	35.1	41.8	56.6	55.4
18:2n-6	5.7	21.5	4.6	5.9	10.3	22.7	23.6
20:3n-6	0.1	0.0	0.1	0.1	0.0	0.0	0.0
20:4n-6	0.1	0.7	0.7	0.7	0.5	0.1	0.1
22:5n-6	0.2	0.0	0.2	0.2	0.2	0.0	0.0
**Σ n-6 PUFA**^4^	7.1	22.1	6.2	7.3	11.4	22.8	23.8
18:3n-3	1.8	7.0	1.4	1.5	4.2	8.7	8.9
20:5n-3	11.9	3.4	10.9	10.4	8.1	0.6	0.5
22:5n-3	1.2	0.2	1.2	1.2	1.0	0.1	0.1
22:6n-3	14.1	2.5	10.5	9.7	8.2	0.8	0.7
**Σ n-3 PUFA**^5^	33.0	14.6	27.9	26.4	24.1	10.4	10.3
**Σ n-3 LC-PUFA**^6^	27.8	6.2	23.4	22.0	17.8	1.5	1.2
**n-3:n-6**	4.6	0.7	4.5	3.6	2.1	0.5	0.4

*MUFA* monounsaturated fatty acid, *PUFA* polyunsaturated fatty acid, *SFA* saturated fatty acid

^1^For further details and sources see McMillan et al. 2024

^2^Contains 15:0, 16:0, 18:0, 20:0, 22:0, and 24:0

^3^Contains 16:1n − 9, 18:1n − 9, 20:1n-11, 20:1n-9, 22:1n-11, 22:1n-9 and 24:1n − 9

^4^Contains 18:3n-6, 20:2n − 6, and 22:4n − 6

^5^Contains 18:4n-3, 20:3n-3, 20:4n-3 and 21:5n-3

^6^n-3 LC-PUFA, the sum of 20:4n-3, 20:5n-3, 22:5n-3 and 22:6n-3

### Sampling

Samples were collected at three time points: S1, after 2 weeks (following completion of the 1- or 2-week stimulus periods); S2, end of the intermediate phase; S3, end of the challenge phase (Fig. 1). At all sampling points, fish were fasted for 24 h and bulk weighed per tank before a selected number of fish were randomly sampled and euthanised with tricaine methanesulfonate (MS-222; 1000 mg/L in hydrogen carbonate-buffered solution). At S1, 40 fish per tank were sampled and weighed, then pooled as one sample, frozen at −20 °C, and immediately freeze-dried (Christ Alpha 1–4 LSC; Osterode am Harz, Germany) prior to biochemical analyses. At the S2 and S3 sampling points, 11 randomly selected fish from each tank were weighed individually; the liver and anterior intestine (1 cm length immediately posterior to the pyloric caeca) were collected from six fish and divided into two portions, with one portion placed in 1.5 mL RNALater (Sigma, Poole, UK) and stored overnight at 4 °C before freezing at − 70 °C prior to molecular analysis. The other portion was placed in 2 mL 4% buffered formalin for subsequent histological analysis. The other 5 sampled fish were dissected and liver, whole intestine, white muscle, gill, brain, and eye were collected and placed immediately into liquid nitrogen and then stored at − 70 °C prior to biochemical analyses.

### Biochemical compositions of diets

Biochemical compositions of experimental diets were determined using the standard procedures of the Association of Official Analytical Chemists (AOAC 2000). Briefly, moisture contents were determined by gravimetric loss based on the thermal drying method by heating in an oven at 105 °C for 16 h. Protein contents were based on analysed N content (*N* × 6.25), determined by the Kjeldahl method using an auto-analyser (Opsis AB LiquidLINE KjelROC; Kjeltech, Furulund, Sweden). Ash contents were determined by incineration in a muffle furnace at 600 °C for 16 h, and energy content was analysed by bomb calorimetry (Autobomb; Gallenkamp & Co. Ltd., Cambridge, UK). However, dietary lipid contents and fatty acid compositions were determined by the same methods used to analyse fish and tissues. Thus, lipid was determined gravimetrically according to Folch et al. (1957), and fatty acid compositions were determined by analysis of fatty acid methyl esters (FAME) by GC as detailed below.

### Lipid content, class composition and fatty acid analyses of fish

Total lipids (TL) of whole fish (S1) and tissues (S2 and S3) were extracted by homogenisation in 20 or 36 mL, respectively, of chloroform/methanol (2:1, v/v) using an Ultra-Turrax tissue disrupter (Fisher Scientific, Loughborough, UK) essentially according to Folch et al. (1957), and lipid content determined gravimetrically. The extracted TL was resuspended at a concentration of 10 mg/mL in chloroform/methanol (2:1) containing 0.01% (w/v) butylated hydroxytoluene (BHT) as antioxidant prior to further analysis.

Lipid classes of whole fish and fish tissues were separated by high-performance thin layer chromatography (HPTLC) on 20 × 10 cm plates (VWR, Lutterworth, UK) according to Henderson and Tocher (1992). The mobile solvent phase consisting of methyl acetate/isopropanol/chloroform/methanol/0.25% (w/v) potassium chloride (25:25:25:10:9, by vol.) was used for the separation of polar lipid classes by developing the solvent to approximately half-distance up the plate, with total neutral lipids running at the solvent front. After drying, the plate was then fully developed using a mobile solvent phase consisting of isohexane/diethyl ether/glacial acetic acid (85:15:1.5, by vol.) for the separation of neutral lipid classes. Lipid classes were visualised after spraying with 3% copper acetate and 8% phosphoric acid and placing in a drying oven at 160 °C for 20 min. The plates were scanned by a TLC Scanner 3 (CAMAG, Muttenz, Switzerland) and data processed using the Wincat software package (CAMAG). Data were presented as relative proportions of triacylglycerol (TAG) and total polar lipids (sum of all polar lipid classes).

Fatty acid methyl esters were prepared from TL of diets, whole fish, and fish tissues by acid-catalysed transmethylation at 50 °C for 16 h and purified as described previously (Christie 2003; Tocher and Harvie 1988). The FAME were separated and quantified by gas–liquid chromatography (Thermo Finnigan Trace GC, Thermo Scientific, Milan, Italy) equipped with a 30 m × 0.32 mm i.d. × 0.25 µm ZB-wax column (Phenomenex, Cheshire, UK), “on column” injection and flame ionisation detection. Hydrogen was used as the carrier gas at constant pressure (175 kPa) with the initial oven thermal gradient from 50 °C to 150 °C at 40 °C.min^−1^, then 195 °C at 2 °C.min^−1^, and 205 °C at 0.5 °C.min^−1^ to a final temperature of 230 °C at 40 °C.min^−1^. Individual FAME were identified by comparison to an in-house marine fish oil (“Marinol”) standard and commercial standards (Restek 20-FAME Marine Oil Standard; Thames Restek UK Ltd., Buckinghamshire, UK). The data were collected and processed using Chromcard data system for Windows (Version 2.11; Thermo Fisher Scientific Inc.). Heptadecanoic acid (17:0) was used as an internal standard to calculate fatty acid content per gram tissue.

For fatty acid compositions of total polar lipids (PL) of fish tissues, lipid classes were first separated by TLC on 20 × 20 cm plates (VWR, Lutterworth, UK) using the respective solvent phases described above. Individual polar lipid classes were identified by spraying plates with 0.1% (w/v) 2–7-dichlorofluorescein in 97% methanol (v/v), marked under UV light at 240 nm (UVP® Mineralight®R-52G; UVP Inc.) and the silica scraped and combined into a single tube (total PL) and subjected to direct methylation on the silica using acid-catalysed transmethylation as above. The total PL FAMEs were extracted and analysed by GC under the same conditions detailed above.

### Molecular analyses

Total RNA of liver of six individual fish per tank was extracted by homogenising in 1 mL of TriReagent® according to the manufacturer’s instructions (Sigma-Aldrich, Dorset, UK). The quantity, quality and concentration of RNA were determined by spectrophotometry (Nanodrop ND-1000; Labtech Int.), and RNA integrity was checked by 1% agarose gel electrophoresis. The liver RNA extracts from each tank were pooled into two samples (three livers per pool, six pools per dietary treatment, *n* = 3), and 2000 ng RNA was used for cDNA synthesis with a random primer in a 20 µL reaction volume using a high-capacity reverse transcription kit following the manufacturer’s protocol (Applied Biosystems, Warrington, UK). The synthesised cDNA was diluted 20-fold with milliQ water (Thermo Fisher Scientific).

Expression levels of genes of interest (GOI) involved in key pathways including LC-PUFA biosynthesis (*elovl2*, *elovl5a*, *elovl5b*, *fads2d5*, and *fads2d6*), transcription factors (*lxr*, *ppara*, *pparg*, *srebp1*, and *srebp2*), and lipid metabolism (*aco*, *cpt1*, *fas*, and *hmgcr*) were determined by real-time quantitative PCR as described in detail by Betancor et al. (2021). Detailed information on GOI, housekeeping genes and primers is shown in Online Resource 1. The qPCR was performed using a qTower^3^ G real-time PCR Thermal Cycler (Analytic Jena GmbH, Jena, Germany) in 96-well plates in duplicate 10 µL reaction volumes containing 5 µL of Luminaris Color Higreen RT-PCR master mix kit (Thermo Scientific, Hemel Hempstead, UK), 1.0 µL of primers of GOI, 1.5 µL of molecular biology grade water, and 2.5 µL of cDNA. For housekeeping genes, 1 µL of cDNA and 3 µL of molecular biology grade water were used. A negative control without cDNA (no template control) was used to ensure no contaminant was present in the master mix. Standard amplification parameters contained a DNase pre-treatment at 50 °C for 2 min, an initial activation step at 95 °C for 10 min, followed by 35 cycles: 15 s at 95 °C, 30 s at the annealing Tm, and 30 s at 72 °C. Results were normalised using the ΔCt method (Pfaffl 2001) using reference housekeeping genes, elongation factor 1 alpha (*ef1α*), hypoxanthine–guanine phosphoribosyltransferase (*hprt*) and ribosomal protein L2 (*rpl2*), which were identified as the most stable according to geNorm (Pattyn et al. 2003).

### Histological analyses

Liver and anterior intestine samples were rinsed in water and fixed in 4% buffered neutral-formaldehyde using a Shandon Citadel 2000 Automatic Tissue Processor (Thermo Scientific, Basingstoke, UK), and then embedded into paraffin blocks (Histo-embedder, Jung, Leica, Germany). Tissue was sectioned in 5 µm thickness wax slices, and all slides stained with Haematoxylin–Eosin, while intestine samples were also stained with Alcian Blue/Periodic Acid Schiff (ABPAS) for acidic and neutral goblet cells. Slides were scanned (Axio Scan.Z1 slide scanner; ZEISS, Cambridge, UK) and the digital images analysed using Qupath v0.3.0 (Bankhead et al. 2017) and ImageJ (Research Services Branch, National Institute of Mental Health, Bethesda, Maryland, USA) software. Data were collected from six randomly chosen fields from each tissue image at 20 × magnification. The following histological factors were measured: intracytoplasmic lipid vacuolisation; height and width of enterocyte; AB and PAS positive goblet cell density; intestinal circular muscle thickness. Measurement details are illustrated in Online Resource 2. The intracytoplasmic lipid vacuolisation was calculated by dividing the lipocyte area by the respective chosen area, and the result was expressed as an amount per 10^5^ μm^2^. Five images of PAS-stained sections were analysed by ImageJ. The area selection tool was used to define the region of interest corresponding to the intestinal villi, and the total villus area in each image was calculated. Within the defined area, a plugin was used to detect the round-stained particles, which were identified as goblet cells. The total number of goblet cells was then measured in each image. Goblet cell density was calculated as the ratio of goblet cell count to the area of intestinal villi in each image. The mean value was based on five measurements. Height and width of the enterocytes, and intestinal circular muscle thickness were determined as described previously (Escaffre et al. 2007).

### Statistical analyses

All data are shown as means ± standard deviation. Percentage data were arcsine square-root transformed before statistical analyses. All variables were tested for normality using the Shapiro–Wilk test, and for homogeneity of variances with Levene’s test. The significance of differences was set at *p* < 0.05. The significance of differences between groups at the same sampling point was determined by one-way analysis of variance (ANOVA) followed by Tukey’s post hoc test for multiple comparisons of means where differences were detected. The significance of differences between sampling points S2 and S3 for liver was determined by a paired *t*-test. Multivariate principal component analysis (PCA), by which the general clustering trends of samples from multi-dimensions can be visualised through dimension reduction, was conducted to define the similarity between groups with the chosen features. All statistical analyses were performed using SPSS software (IBM SPSS Statistics 19; SPSS Inc., Chicago, IL, USA) and GraphPad Prism 8 (GraphPad Software, USA).

## Results

### Growth performance

At the end of the stimulus phase (first 2 weeks after first feeding), fish from all experimental groups had doubled in weight, although the M group revealed higher final weight and specific growth rate than the V1 and V2 groups (Table 2). However, fish weights at the end of the intermediate and challenge phases showed no significant differences between groups. No differences in feed intake or feed efficiency were observed among groups at the end of the feeding trial. Full details of growth performance, nutrient retentions, and other performance-related parameters are presented elsewhere (McMillan et al. 2024).

**Table 2:** Growth performance of Atlantic salmon for each phase of the study

	*M*	V1	V2
**Whole fish weight (g)**			
End of stimulus phase	0.36 ± 0.02^a^	0.33 ± 0.01^b^	0.34 ± 0.00^b^
End of intermediate phase	15.24 ± 0.24	15.73 ± 0.54	14.46 ± 0.64
End of challenge phase	33.12 ± 2.10	35.88 ± 2.67	31.78 ± 4.34
**Biomass gain (kg)**			
Stimulus phase	0.05 ± 0.00	0.05 ± 0.00	0.05 ± 0.00
Challenge phase	2.76 ± 0.31	2.96 ± 0.32	2.67 ± 0.53
**Specific growth rate (SGR, %/day)**^**1**^			
Stimulus phase	6.29 ± 0.23^a^	5.98 ± 0.27^b^	5.94 ± 0.06^b^
Challenge phase	1.82 ± 0.12	1.93 ± 0.15	1.84 ± 0.23
**Voluntary feed intake (VFI, kg)**^**2**^			
Stimulus phase	0.04 ± 0.00	0.02 ± 0.00	0.04 ± 0.00
Challenge phase	2.67 ± 0.01	2.63 ± 0.02	2.62 ± 0.04
**Feed efficiency (FE)**^**3**^			
Stimulus phase	1.6 ± 0.1	2.0 ± 0.1	1.5 ± 0.1
Challenge phase	1.0 ± 0.1	1.1 ± 0.1	1.0 ± 0.1

Data are means of triplicate tanks with standard deviations (*n* = 3). A *p*-value of less than 0.05 is considered statistically significant. Superscript letters denote significant differences between dietary groups within a row

^1^Specific growth rate (SGR) = 100 × (ln*W*_*t*_ − ln*W*_*i*_)/*t*. For each phase, *W*_*t*_ refers to final weight; *W*_*i*_ refers to initial weight; *t* refers to the duration in days

^2^Voluntary feed intake (VFI) = total feed supplied − total uneaten feed. The VFI data were calculated for both stimulus and challenge phases

^3^Feed efficiency (FE) = biomass gain/total feed intake

### Tissue lipid contents and lipid classes

**Fig. 2:**
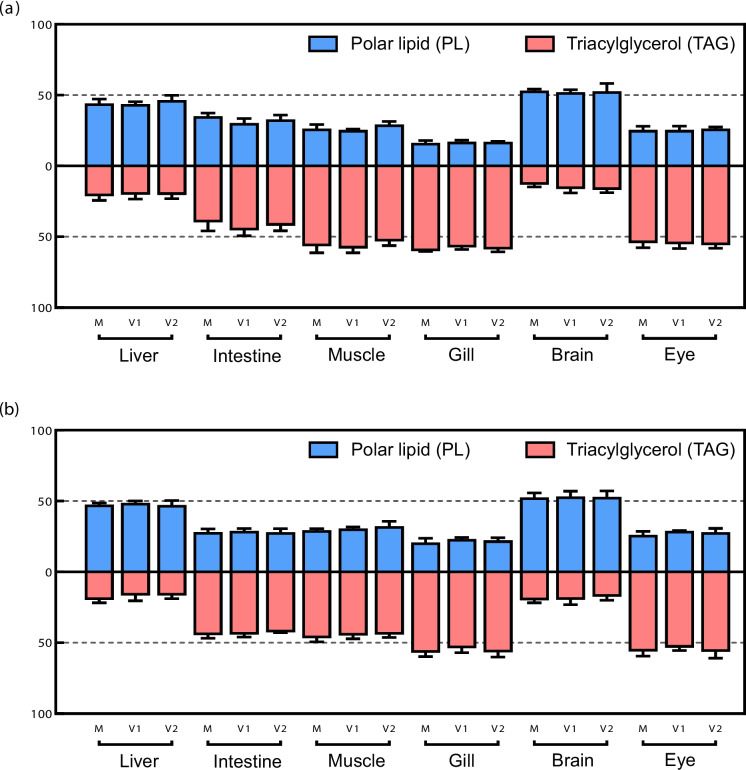
The proportions of total polar lipids and triacylglycerol of Atlantic salmon tissues among groups at the end of the intermediate phase (**a**) and after the challenge phase (**b**). Data are means of three replicates (*n* = 3), with standard deviation represented by vertical bars. There were no significant differences between dietary groups within each tissue at either timepoint

No significant differences in lipid contents were found between dietary groups (V1, V2 and M) in either whole fish after the stimulus phase (Table 3), or in liver at the end of the intermediate and challenge phases (Table 4), or in any of the other analysed tissues at the end of the challenge phase (Tables 5 and 6). Dietary regime during the stimulus phase had little influence on the lipid class composition of any of the analysed tissues, with no differences detected in relative proportions of PL and TAG among dietary groups (Fig. 2). In addition, the similar levels of PL and TAG proportions in all tissues pre- (end of intermediate phase) and post-challenge indicated that proportions were not affected by the change in diet from M to V. Among the tissues, PL were highest in liver and brain (45% and 53% of TL, respectively), while TAG was the predominant lipid class in the other tissues (Fig. 2).

**Table 3:** Lipid content and fatty acid compositions (percentage of total fatty acids) of total lipid of whole Atlantic salmon after the stimulus phase

	*M*	V1	V2
**Lipid content**	2.7 ± 0.2	2.6 ± 0.2	2.7 ± 0.1
**Σ SFA**^1^	26.9 ± 0.5^a^	25.5 ± 0.4^b^	20.2 ± 0.3^c^
**Σ MUFA**^2^	30.5 ± 0.9^b^	31.0 ± 0.2^b^	37.8 ± 0.4^a^
18:2n-6	6.2 ± 0.2^c^	7.6 ± 0.2^b^	14.1 ± 0.1^a^
20:3n-6	0.3 ± 0.0^b^	0.4 ± 0.0^b^	0.8 ± 0.0^a^
20:4n-6 (ARA)	1.4 ± 0.1^a^	1.4 ± 0.0^a^	1.2 ± 0.0^b^
22:5n-6	0.2 ± 0.0	0.2 ± 0.0	0.2 ± 0.0
**Σ n-6 PUFA**^3^	8.9 ± 0.3^c^	10.4 ± 0.2^b^	17.7 ± 0.2^a^
18:3n-3	2.0 ± 0.1^b^	2.3 ± 0.1^b^	3.6 ± 0.2^a^
20:5n-3 (EPA)	7.4 ± 0.2^a^	7.0 ± 0.1^a^	4.5 ± 0.1^c^
22:5n-3	3.0 ± 0.1^a^	3.2 ± 0.1^a^	2.6 ± 0.0^b^
22:6n-3 (DHA)	17.8 ± 0.9^a^	17.1 ± 0.1^a^	10.6 ± 0.2^b^
**Σ n-3 PUFA**^4^	33.3 ± 1.1^a^	32.6 ± 0.5^a^	23.9 ± 0.4^b^
**Σ n-3 LC-PUFA**^5^	29.7 ± 1.2^a^	28.9 ± 0.4^a^	19.1 ± 0.3^b^
**n-3:n-6**	3.4 ± 0.2^a^	3.1 ± 0.1^b^	1.3 ± 0.0^c^

Data are means of triplicate tanks with standard deviations (*n* = 3). Different superscript letters within a row denote significant differences among groups determined by one-way ANOVA with Tukey’s comparison test (*p* < 0.05)

*MUFA* monounsaturated fatty acid, *PUFA* polyunsaturated fatty acid, *SFA* saturated fatty acid

^1^Contains 15:0, 16:0, 18:0, 20:0, 22:0 and 24:0

^2^Contains 16:1n − 9, 18:1n − 9, 20:1n-11, 20:1n-9, 22:1n-11, 22:1n-9 and 24:1n − 9

^3^Contains 18:3n-6, 20:2n − 6, 20:3n − 6, 22:4n − 6 and 22:5n − 6

^4^Contains 18:4n-3, 20:3n-3, 20:4n-3, 21:5n-3 and 22:5n-3

^5^n-3 LC-PUFA, the sum of 20:4n-3, 20:5n-3, 22:5n-3 and 22:6n-3

**Table 4:** Fatty acid compositions of total lipid and total polar lipids (percentage of fatty acids) of liver at the end of intermediate and challenge phases

	End of intermediate phase			End of challenge phase			
	*M*	V1	V2	*M*	V1	V2	
Lipid % (wet wt.)	4.6 ± 0.4	4.9 ± 0.4	5.0 ± 0.6	4.7 ± 0.5	5.3 ± 0.7	5.5 ± 0.7	
**Total lipid FA %**							
**Σ SFA**^1^	23.2 ± 0.4	22.4 ± 0.6	22.6 ± 0.9	19.6 ± 1.1	20.8 ± 0.6	20.7 ± 0.9	
**Σ MUFA**^2^	33.4 ± 2.1	36.2 ± 2.8	35.5 ± 3.5	43.8 ± 3.8	40.5 ± 3.2	40.2 ± 3.0	
18:2n-6	5.6 ± 0.6	6.1 ± 0.7	6.0 ± 0.7	8.4 ± 0.6*	8.4 ± 0.2*	8.6 ± 0.6*	
20:3n-6	0.7 ± 0.1	0.7 ± 0.0	0.7 ± 0.0	2.3 ± 0.7*	3.1 ± 0.3*	2.7 ± 0.6*	
20:4n-6 (ARA)	2.0 ± 0.3	1.9 ± 0.2	1.9 ± 0.2	4.9 ± 0.7*	5.4 ± 0.6*	5.2 ± 0.6*	
22:5n-6	0.3 ± 0.0	0.3 ± 0.0	0.3 ± 0.1	1.1 ± 0.2*	1.3 ± 0.1*	1.5 ± 0.1*	
**Σ n-6 PUFA**^3^	9.6 ± 0.1	10.2 ± 0.8	10.1 ± 0.5	19.0 ± 0.9*	20.7 ± 0.5*	20.5 ± 0.9*	
18:3n-3	1.4 ± 0.3	1.3 ± 0.1	1.3 ± 0.1	1.2 ± 0.1	1.1 ± 0.1	1.0 ± 0.0	
20:5n-3 (EPA)	4.1 ± 0.3	3.9 ± 0.3	3.9 ± 0.5	1.7 ± 0.8*	1.8 ± 0.3*	1.8 ± 0.0*	
22:5n-3	1.1 ± 0.1	0.9 ± 0.1	0.9 ± 0.1	0.9 ± 0.3	1.0 ± 0.2	0.7 ± 0.2	
22:6n-3 (DHA)	25.7 ± 3.4	23.9 ± 2.7	24.3 ± 2.3	12.4 ± 2.1*	13.0 ± 2.0*	13.6 ± 2.3*	
**Σ n-3 PUFA**^4^	33.4 ± 2.7	31.0 ± 2.9	31.1 ± 2.8	17.4 ± 3.5*	17.8 ± 2.5*	18.3 ± 2.6*	
**Σ n-3 LC-PUFA**^5^	31.5 ± 3.4	29.3 ± 3.1	29.5 ± 2.9	15.3 ± 5.2*	15.9 ± 2.4*	16.6 ± 2.5*	
**n-3:n-6**	3.5 ± 0.3	3.1 ± 0.5	3.1 ± 0.6	0.9 ± 0.3*	0.9 ± 0.1*	0.9 ± 0.2*	
**Polar lipid FA %**							
**Σ SFA**^1^	28.4 ± 0.3	27.5 ± 0.7	27.8 ± 0.1	24.0 ± 1.1	24.4 ± 0.6	24.1 ± 0.6	
**Σ MUFA**^2^	19.4 ± 0.5	20.4 ± 0.6	19.6 ± 0.7	26.3 ± 2.8*	25.7 ± 1.8*	26.3 ± 2.4*	
18:2n-6	3.2 ± 0.0	3.4 ± 0.1	3.4 ± 0.1	5.8 ± 1.0*	6.2 ± 0.3*	5.9 ± 0.3*	
20:3n-6	0.8 ± 0.1	0.7 ± 0.0	0.8 ± 0.0	3.1 ± 1.1*	4.1 ± 0.3*	3.5 ± 0.8*	
20:4n-6	3.1 ± 0.1	3.1 ± 0.5	3.1 ± 0.1	8.9 ± 1.2*	8.7 ± 0.2*	9.3 ± 0.3*	
22:5n-6	0.4 ± 0.0	0.4 ± 0.0	0.4 ± 0.0	2.2 ± 0.4*	2.4 ± 0.1*	2.4 ± 0.1*	
**Σ n-6 PUFA**^3^	8.3 ± 0.1	8.1 ± 0.1	8.1 ± 0.3	21.6 ± 2.4*	23.1 ± 0.1*	23.0 ± 1.5*	
18:3n-3	0.6 ± 0.0	0.6 ± 0.0	0.6 ± 0.0	0.6 ± 0.1	0.6 ± 0.1	0.6 ± 0.0	
20:5n-3	4.6 ± 0.4	4.8 ± 0.1	4.7 ± 0.2	2.6 ± 0.2*	2.5 ± 0.1*	2.4 ± 0.1*	
22:5n-3	1.0 ± 0.1	1.0 ± 0.0	1.0 ± 0.1	1.4 ± 0.2	1.5 ± 0.2	1.2 ± 0.2	
22:6n-3	36.9 ± 0.8	36.9 ± 0.4	37.3 ± 0.2	22.5 ± 1.7*	21.8 ± 1.2*	21.1 ± 2.5*	
**Σ n-3 PUFA**^4^	43.7 ± 0.4	43.8 ± 0.3	44.2 ± 0.3	27.9 ± 2.1*	26.8 ± 1.3*	26.3 ± 2.6*	
**Σ n-3 LC-PUFA**^5^	42.9 ± 0.4	43.0 ± 0.3	43.4 ± 0.2	26.8 ± 2.2*	25.8 ± 1.3*	25.3 ± 2.6*	
**n-3:n-6**	5.3 ± 0.1	5.4 ± 0.1	5.4 ± 0.2	1.3 ± 0.2*	1.2 ± 0.1*	1.2 ± 0.2*	

Data are means of triplicate tanks with standard deviations (*n* = 3). There were no significant differences among treatments at the end of each phase. Asterisks (*) denote significant differences between the end of the intermediate phase and the end of the challenge phase for each dietary treatment (*p* < 0.05)

*FA* fatty acid, *MUFA* monounsaturated fatty acid, *PUFA* polyunsaturated fatty acid, *SFA* saturated fatty acid

^1^Contains 15:0, 16:0, 18:0, 20:0, 22:0 and 24:0

^2^Contains 16:1n − 9, 18:1n − 9, 20:1n-11, 20:1n-9, 22:1n-11, 22:1n-9 and 24:1n − 9

^3^Contains 18:3n-6, 20:2n − 6, and 22:4n − 6

^4^Contains 18:4n-3, 20:3n-3, 20:4n-3 and 21:5n-3

^5^n-3 LC-PUFA, the sum of 20:4n-3, 20:5n-3, 22:5n-3 and 22:6n-3

**Table 5:** Fatty acid compositions of total lipid and total polar lipids (percentage of fatty acids) of intestine, muscle and gill at the end of challenge phase

	Intestine			Muscle			Gill		
	*M*	V1	V2	*M*	V1	V2	*M*	V1	V2
Lipid % (wet wt.)	9.7 ± 0.4	9.3 ± 0.3	8.9 ± 1.0	6.9 ± 0.6	6.5 ± 0.5	6.1 ± 0.4	10.6 ± 1.0	10.4 ± 0.7	10.3 ± 0.5
**Total lipid FA %**									
**Σ SFA**^1^	19.8 ± 1.6	19.4 ± 0.5	19.4 ± 0.4	18.2 ± 0.4	17.7 ± 0.1	17.8 ± 0.2	20.1 ± 0.2	19.3 ± 0.4	19.3 ± 0.2
**Σ MUFA**^2^	47.0 ± 0.8	46.9 ± 0.9	46.7 ± 1.1	47.9 ± 1.2	48.3 ± 0.1	47.7 ± 0.2	48.4 ± 0.1	48.6 ± 0.7	48.5 ± 0.5
18:2n-6	12.9 ± 1.0	13.2 ± 0.4	12.8 ± 0.3	13.4 ± 0.5	13.9 ± 0.2	13.7 ± 0.1	12.6 ± 0.4	13.1 ± 0.2	12.9 ± 0.3
20:3n-6	1.2 ± 0.2	1.3 ± 0.1	1.3 ± 0.1	1.1 ± 0.2	1.2 ± 0.1	1.2 ± 0.1	0.9 ± 0.1	0.9 ± 0.1	0.9 ± 0.1
20:4n-6	1.4 ± 0.0	1.4 ± 0.0	1.6 ± 0.2	0.8 ± 0.1	0.8 ± 0.0	0.8 ± 0.0	1.1 ± 0.0	1.1 ± 0.0	1.1 ± 0.1
22:5n-6	0.5 ± 0.1	0.5 ± 0.1	0.6 ± 0.0	0.3 ± 0.0	0.3 ± 0.0	0.3 ± 0.0	0.3 ± 0.1	0.3 ± 0.0	0.3 ± 0.0
**Σ n-6 PUFA**^3^	18.2 ± 1.4	18.5 ± 0.6	18.4 ± 0.2	17.7 ± 1.0	18.5 ± 0.3	18.2 ± 0.3	16.7 ± 0.9	17.3 ± 0.3	17.1 ± 0.5
18:3n-3	3.2 ± 0.2	3.1 ± 0.1	3.0 ± 0.1	3.5 ± 0.1	3.5 ± 0.1	3.5 ± 0.1	3.3 ± 0.0	3.4 ± 0.0	3.3 ± 0.1
20:5n-3	1.4 ± 0.1	1.6 ± 0.1	1.6 ± 0.1	1.7 ± 0.0	1.6 ± 0.1	1.8 ± 0.0	1.6 ± 0.1	1.6 ± 0.1	1.7 ± 0.1
22:5n-3	0.7 ± 0.1	0.7 ± 0.1	0.7 ± 0.0	0.8 ± 0.1	0.7 ± 0.0	0.7 ± 0.0	0.7 ± 0.1	0.7 ± 0.1	0.7 ± 0.1
22:6n-3	7.1 ± 0.8	7.1 ± 0.6	7.6 ± 0.7	7.3 ± 0.6	7.0 ± 0.2	7.4 ± 0.1	6.3 ± 0.4	6.3 ± 0.5	6.3 ± 0.4
**Σ n-3 PUFA**^4^	14.4 ± 0.7	14.5 ± 0.9	14.8 ± 0.9	15.8 ± 1.2	15.2 ± 0.2	15.9 ± 0.2	14.2 ± 0.7	14.2 ± 0.7	14.4 ± 0.7
**Σ n-3 LC-PUFA**^5^	9.7 ± 1.0	9.9 ± 0.8	10.4 ± 0.9	10.3 ± 1.0	9.9 ± 0.3	10.6 ± 0.2	9.2 ± 0.6	9.1 ± 0.7	9.4 ± 0.6
**n-3:n-6**	0.8 ± 0.1	0.8 ± 0.1	0.8 ± 0.1	0.9 ± 0.1	0.8 ± 0.0	0.9 ± 0.0	0.9 ± 0.1	0.8 ± 0.1	0.8 ± 0.1
**Polar lipid FA %**									
**Σ SFA**^1^	28.8 ± 1.6	28.4 ± 1.1	28.2 ± 0.4	20.1 ± 0.6	19.8 ± 0.2	20.1 ± 0.3	27.6 ± 0.3	27.2 ± 0.6	27.7 ± 0.6
**Σ MUFA**^2^	24.3 ± 1.9	25.4 ± 3.1	25.2 ± 1.1	24.2 ± 1.9	24.6 ± 0.2	24.3 ± 0.3	27.5 ± 0.5	26.8 ± 0.3	27.3 ± 1.3
18:2n-6	7.5 ± 0.7	8.0 ± 0.6	7.9 ± 0.2	8.9 ± 0.9	9.5 ± 0.3	9.1 ± 0.1	5.1 ± 0.2	5.9 ± 0.2	5.2 ± 0.5
20:3n-6	3.3 ± 0.6	3.5 ± 0.3	3.4 ± 0.5	2.7 ± 0.4	2.9 ± 0.2	2.8 ± 0.2	2.5 ± 0.4	2.9 ± 0.1	2.5 ± 0.5
20:4n-6	6.5 ± 0.1	6.1 ± 0.3	6.4 ± 0.4	3.3 ± 0.2	3.2 ± 0.2	3.2 ± 0.2	6.6 ± 0.2	7.0 ± 0.3	7.1 ± 0.9
22:5n-6	2.6 ± 0.8	2.2 ± 0.4	2.4 ± 0.2	1.4 ± 0.1	1.4 ± 0.1	1.4 ± 0.1	1.6 ± 0.2	1.8 ± 0.1	1.7 ± 0.2
**Σ n-6 PUFA**^3^	21.6 ± 2.1	21.5 ± 1.7	21.5 ± 1.2	18.2 ± 1.9	18.9 ± 0.8	18.3 ± 0.6	17.0 ± 1.3	19.2 ± 0.5	17.6 ± 2.6
18:3n-3	0.7 ± 0.3	0.8 ± 0.3	0.8 ± 0.2	2.7 ± 0.1	3.0 ± 0.0	2.9 ± 0.1	0.8 ± 0.1	0.8 ± 0.1	0.8 ± 0.0
20:5n-3	1.6 ± 0.2	1.6 ± 0.1	1.5 ± 0.0	4.2 ± 0.5	4.0 ± 0.2	4.2 ± 0.1	2.3 ± 0.3	2.2 ± 0.0	2.2 ± 0.1
22:5n-3	0.7 ± 0.0	0.7 ± 0.0	0.7 ± 0.0	1.5 ± 0.1	1.4 ± 0.1	1.5 ± 0.0	0.9 ± 0.0	0.8 ± 0.0	0.9 ± 0.0
22:6n-3	19.9 ± 1.8	19.1 ± 2.7	19.5 ± 0.6	24.8 ± 0.8	24.2 ± 0.8	24.8 ± 0.7	18.8 ± 0.9	18.4 ± 0.1	18.6 ± 0.8
**Σ n-3 PUFA**^4^	23.5 ± 1.6	22.8 ± 2.7	23.2 ± 0.7	35.5 ± 3.2	34.9 ± 1.0	35.4 ± 0.7	23.3 ± 1.3	22.9 ± 0.1	23.0 ± 0.9
**Σ n-3 LC-PUFA**^5^	22.4 ± 1.6	21.6 ± 2.8	22.0 ± 0.7	31.3 ± 3.4	30.4 ± 1.1	31.2 ± 0.7	22.2 ± 1.2	21.7 ± 0.1	21.9 ± 1.0
**n-3:n-6**	1.1 ± 0.0	1.1 ± 0.1	1.1 ± 0.1	2.0 ± 0.3	1.9 ± 0.1	1.9 ± 0.1	1.4 ± 0.2	1.2 ± 0.0	1.3 ± 0.3

Data are means of triplicate tanks with standard deviations (*n* = 3). A *p*-value of less than 0.05 is considered statistically significant

*FA* fatty acid, *MUFA* monounsaturated fatty acid, *PUFA* polyunsaturated fatty acid, *SFA* saturated fatty acid

^1^Contains 15:0, 16:0, 18:0, 20:0, 22:0 and 24:0

^2^Contains 16:1n − 9, 18:1n − 9, 20:1n-11, 20:1n-9, 22:1n-11, 22:1n-9 and 24:1n − 9

^3^Contains 18:3n-6, 20:2n − 6, and 22:4n − 6

^4^Contains 18:4n-3, 20:3n-3, 20:4n-3 and 21:5n-3

^5^n-3 LC-PUFA, the sum of 20:4n-3, 20:5n-3, 22:5n-3 and 22:6n-3

**Table 6:** Fatty acid compositions of total lipid and total polar lipids (percentage of fatty acids) of the brain and eye at the end of challenge phase

	Brain			Eye		
	*M*	V1	V2	*M*	V1	V2
Lipid % (wet wt.)	7.1 ± 0.5	8.1 ± 0.9	6.8 ± 0.1	8.7 ± 0.3	9.0 ± 0.8	7.8 ± 0.2
**Total lipid FA %**						
**Σ SFA**^1^	24.1 ± 2.6	23.6 ± 2.1	24.8 ± 0.7	18.8 ± 0.9	18.4 ± 0.2	18.6 ± 0.2
**Σ MUFA**^2^	37.9 ± 3.2	39.4 ± 3.3	36.3 ± 0.8	48.6 ± 1.1	48.8 ± 0.3	49.0 ± 0.1
18:2n-6	5.2 ± 1.6	5.4 ± 1.1	3.9 ± 0.5	12.6 ± 1.0	13.1 ± 0.3	12.9 ± 0.3
20:3n-6	0.8 ± 0.1	0.8 ± 0.0	0.7 ± 0.1	0.9 ± 0.1	1.0 ± 0.1	0.9 ± 0.1
20:4n-6	1.6 ± 0.4	1.6 ± 0.3	1.8 ± 0.1	0.8 ± 0.0	0.8 ± 0.0	0.8 ± 0.0
22:5n-6	0.4 ± 0.1	0.4 ± 0.1	0.4 ± 0.0	0.2 ± 0.0	0.3 ± 0.0	0.2 ± 0.0
**Σ n-6 PUFA**^3^	8.9 ± 1.7	9.1 ± 1.5	7.5 ± 0.8	16.6 ± 1.4	17.4 ± 0.4	16.9 ± 0.3
18:3n-3	1.3 ± 0.7	1.3 ± 0.8	0.9 ± 0.3	3.3 ± 0.2	3.3 ± 0.1	3.4 ± 0.1
20:5n-3	3.0 ± 0.4	2.9 ± 0.6	3.2 ± 0.3	1.7 ± 0.3	1.5 ± 0.1	1.6 ± 0.1
22:5n-3	1.3 ± 0.2	1.2 ± 0.2	1.4 ± 0.1	0.7 ± 0.2	0.6 ± 0.0	0.7 ± 0.0
22:6n-3	19.2 ± 2.2	18.1 ± 1.7	21.3 ± 1.4	7.4 ± 1.2	7.4 ± 0.4	7.2 ± 0.2
**Σ n-3 PUFA**^4^	25.8 ± 2.5	24.4 ± 2.2	27.5 ± 1.3	15.5 ± 1.5	15.0 ± 0.5	15.1 ± 0.1
**Σ n-3 LC-PUFA**^5^	23.9 ± 2.7	22.5 ± 2.4	26.3 ± 2.7	10.4 ± 1.8	10.0 ± 0.6	10.0 ± 0.2
**n-3:n-6**	3.1 ± 1.1	3.1 ± 1.7	3.8 ± 0.9	0.9 ± 0.2	0.9 ± 0.0	0.9 ± 0.0
**Polar lipid FA %**						
**Σ SFA**^1^	28.1 ± 0.5	27.4 ± 0.4	27.7 ± 1.0	25.0 ± 0.2	24.8 ± 0.1	24.3 ± 0.6
**Σ MUFA**^2^	32.2 ± 0.5	32.4 ± 0.9	31.8 ± 2.0	24.7 ± 0.5	24.5 ± 0.7	26.9 ± 0.8
18:2n-6	0.9 ± 0.1	0.9 ± 0.2	0.9 ± 0.2	3.4 ± 0.7	3.4 ± 0.3	3.5 ± 0.2
20:3n-6	0.7 ± 0.3	0.7 ± 0.3	0.7 ± 0.1	1.6 ± 0.4	1.7 ± 0.1	1.6 ± 0.2
20:4n-6	1.9 ± 0.1	1.9 ± 0.1	1.9 ± 0.1	3.4 ± 0.4	3.4 ± 0.2	3.3 ± 0.1
22:5n-6	0.5 ± 0.1	0.5 ± 0.0	0.5 ± 0.0	1.1 ± 0.3	1.2 ± 0.1	1.1 ± 0.1
**Σ n-6 PUFA**^3^	4.3 ± 0.3	4.4 ± 0.2	4.3 ± 0.4	10.2 ± 2.0	10.5 ± 0.6	10.4 ± 0.6
18:3n-3	0.1 ± 0.0	0.1 ± 0.0	0.2 ± 0.0	0.7 ± 0.1	0.7 ± 0.1	0.7 ± 0.0
20:5n-3	3.3 ± 0.1	3.3 ± 0.1	3.3 ± 0.0	3.5 ± 0.2	3.3 ± 0.1	3.4 ± 0.0
22:5n-3	1.6 ± 0.0	1.6 ± 0.1	1.6 ± 0.0	1.2 ± 0.1	1.1 ± 0.0	1.1 ± 0.0
22:6n-3	24.7 ± 0.6	24.8 ± 0.8	25.2 ± 1.3	32.0 ± 1.6	32.6 ± 1.4	30.3 ± 0.3
**Σ n-3 PUFA**^4^	30.1 ± 0.8	30.2 ± 0.8	30.7 ± 1.2	38.0 ± 1.7	38.3 ± 1.1	36.2 ± 0.3
**Σ n-3 LC-PUFA**^5^	29.8 ± 0.8	30.0 ± 0.8	30.4 ± 1.3	35.1 ± 1.9	35.4 ± 1.2	35.2 ± 0.3
**n-3:n-6**	7.0 ± 0.6	6.8 ± 0.3	7.1 ± 0.4	3.7 ± 0.3	3.6 ± 0.3	3.4 ± 0.2

Data are means of triplicate tanks with standard deviations (n = 3). A p-value of less than 0.05 is considered statistically significant

*FA* fatty acid, *MUFA* monounsaturated fatty acid, *PUFA* polyunsaturated fatty acid, *SFA* saturated fatty acid

^1^Contains 15:0, 16:0, 18:0, 20:0, 22:0 and 24:0

^2^Contains 16:1n − 9, 18:1n − 9, 20:1n-11, 20:1n-9, 22:1n-11, 22:1n-9 and 24:1n − 9

^3^Contains 18:3n-6, 20:2n − 6, and 22:4n − 6

^4^Contains 18:4n-3, 20:3n-3, 20:4n-3 and 21:5n-3

^5^n-3 LC-PUFA, the sum of 20:4n-3, 20:5n-3, 22:5n-3 and 22:6n-3

### Tissue total and polar lipid fatty acid compositions

**Fig. 3:**
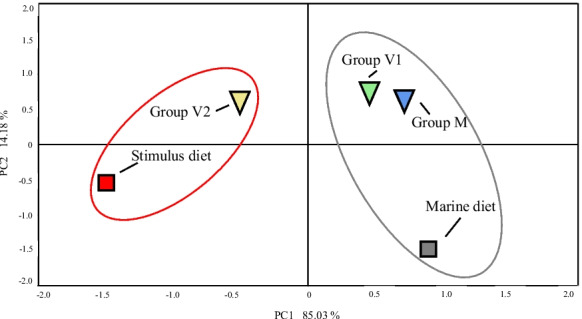
Principal component analysis (PCA) plot based on logarithmically transformed fatty acid compositions of whole fish (V1, V2 and M groups) at the end of the stimulus phase, and the marine and vegetable (stimulus) diets used at the first feeding

At the end of the stimulus phase, the fatty acid compositions of whole fish displayed significant differences among groups (Table 3). Specifically, the data showed that 1 week of feeding with the V diet (V1) was only partially reflected in the fatty acid composition of the fry. Thus, while the percentages of LA and ALA were increased significantly compared to the M group, the proportions of EPA and DHA exhibited no significant differences. However, 2 weeks of feeding the V stimulus diet (V2) had major effects and, compared to the M group, all n-3 LC-PUFA and saturated fatty acids were reduced significantly, while monounsaturated fatty acids and n-6 PUFA, except for ARA, increased significantly (Table 3). Overall, PCA of the fatty acid compositions of whole fish at the end of the stimulus phase revealed that the M and V1 groups were more similar and clustered together with the M marine diet, whereas V2 was separated and clustered with the V vegetable diet (Fig. 3). No differences in fatty acid compositions of either TL or total PL were detected between the dietary groups in liver at the end of either the intermediate or challenge phases (Table 4). Similarly, no differences in fatty acid compositions of TL or total PL were observed between the dietary groups in any of the other analysed tissues at the end of the challenge phase (Tables 5 and 6). In contrast, comparison of liver of fish sampled pre-challenge (S2) and post-challenge (S3) revealed that proportions of all n-6 PUFA in both TL and total PL were elevated significantly by the challenge V diet, whereas EPA and DHA together with the n-3 PUFA: n-6 PUFA declined significantly in all three dietary groups (Table 4). The fatty acid compositions of TL and PL of all other analysed tissues were affected similarly by the change in diet from M to V in the challenge phase (Data not shown).

### Liver gene expression

**Fig. 4:**
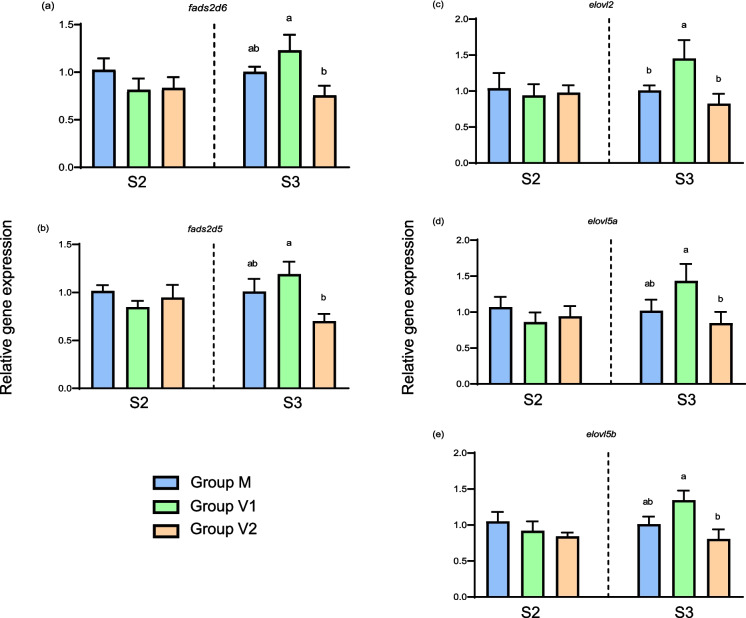
Relative expression levels of genes of long-chain polyunsaturated biosynthesis in the liver of Atlantic salmon. Columns represent mean values (*n* = 3) for the three dietary groups, with standard deviation represented by vertical bars. Data are presented as normalised expression ratios with levels expressed relative to expression in the M group, which was set at 1. Lowercase letters above bars denote significant differences (*p* < 0.05) among dietary groups for expression of each gene at the end of the intermediate (S2) or challenge (S3) phases

Regarding genes of LC-PUFA biosynthesis, at the end of the intermediate phase (S2), no differences were found between any of the dietary groups in the hepatic expression levels of *elovl2, elovl5a, elovl5b, fadsd5* or *fadsd6* (Fig. 4). However, after challenge, the expression levels of both the *fads* and *elovl* genes were significantly higher in V1 fish compared to V2 fish, with levels in the M fish being generally intermediate (Fig. 4).

**Fig. 5:**
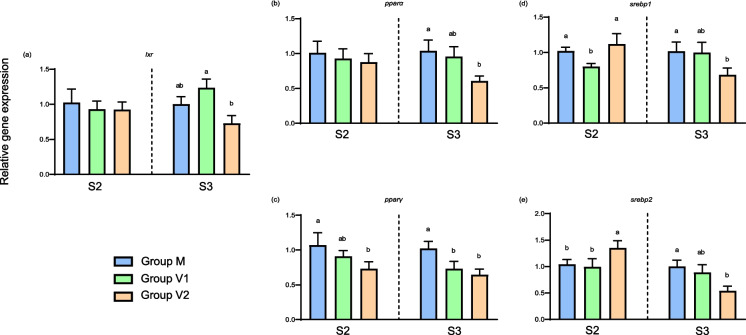
Relative expression levels of genes of transcription factors in the liver of Atlantic salmon. Columns represent mean values (*n* = 3) for the three dietary groups, with standard deviation represented by vertical bars. Data are presented as normalised expression ratios, with levels expressed relative to expression in the M group, which was set at 1. Lowercase letters above bars denote significant differences (*p* < 0.05) among dietary groups for the expression of each gene at the end of the intermediate (S2) or challenge (S3) phases

As for transcription factors, while there were no differences between the dietary groups in expression levels of *lxr* and *pparα* at the end of the intermediate phase, expression levels of *lxr* and *pparα* at the end of the challenge phase were significantly lower in V2 fish compared to V1 and M fish (Fig. 5). Expression levels of *pparγ* were significantly lower in V2 fish than in M fish at the end of both the intermediate and challenge phases, while levels in V1 fish were also lower than M fish at the end of the challenge phase (Fig. 5). The *srebp* regulatory genes showed similar patterns of expression, with levels of both *srebp1* and *srebp2* being highest at the end of the intermediate phase in V2 fish; while, in contrast, expression levels of both genes at the end of the challenge phase were significantly lower in V2 compared to M fish, with levels in V1 fish being intermediate (Fig. 5).

**Fig. 6:**
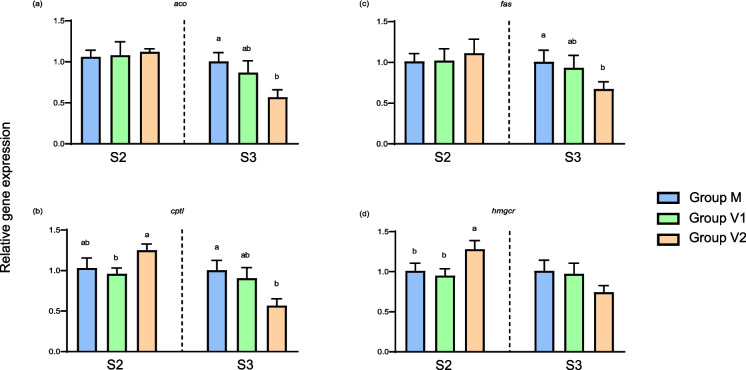
Relative expression levels of genes of lipid metabolism in the liver of Atlantic salmon. Columns represent mean values (*n* = 3) for the three dietary groups, with standard deviation represented by vertical bars. Data are presented as normalised expression ratios, with levels expressed relative to expression in the M group, which was set at 1. Lowercase letters above bars denote significant differences (*p* < 0.05) among dietary groups for the expression of each gene at the end of the intermediate (S2) or challenge (S3) phases

**Fig. 7:**
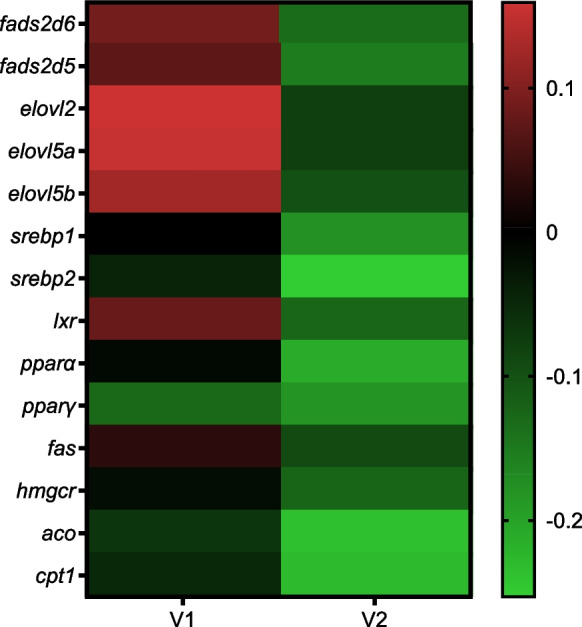
Heatmap showing the expression levels in liver of fourteen target genes at the end of the challenge phase. Levels in the V1 and V2 groups were expressed relative to expression in the M group (set at 1), with columns representing mean values (*n* = 3) for the two groups subjected to the V diet stimulus at first feeding, with each row representing an individual gene. Mean relative expression levels are depicted by a colour scale, indicating low (green), neutral (black) or high (red) relative expression levels, as indicated by the colour bar on the right

The expression of other key genes related to lipid metabolism tended to share a similar pattern (Fig. 6). Thus, at the end of the intermediate phase, expression levels were generally highest in the V2 group, significantly so in the cases of *cpt1 *and* hmgcr* (Fig. 6). In contrast, at the end of the challenge phase, the expression of all the genes displayed the opposite trend so that expression was generally lowest in the V2 group, being significantly lower than levels in the M group, with intermediate levels of expression observed in the V1 group. Although not significant, the expression of *hmgcr* exhibited a similar pattern (*p* = 0.07) (Fig. 6). Comparison of the expression of hepatic genes in V1 and V2 fish relative to the expression levels in M fish at the end of the challenge phase is shown graphically in the heat map in Fig. 7. This demonstrated that the main trends with gene expression levels in fish fed the stimulus V diet for 1 (V1) and 2 weeks (V2) were generally upregulation and down-regulation, respectively, in comparison to “unstimulated” fish fed the M diet at first feeding.

### Histology

**Fig. 8:**
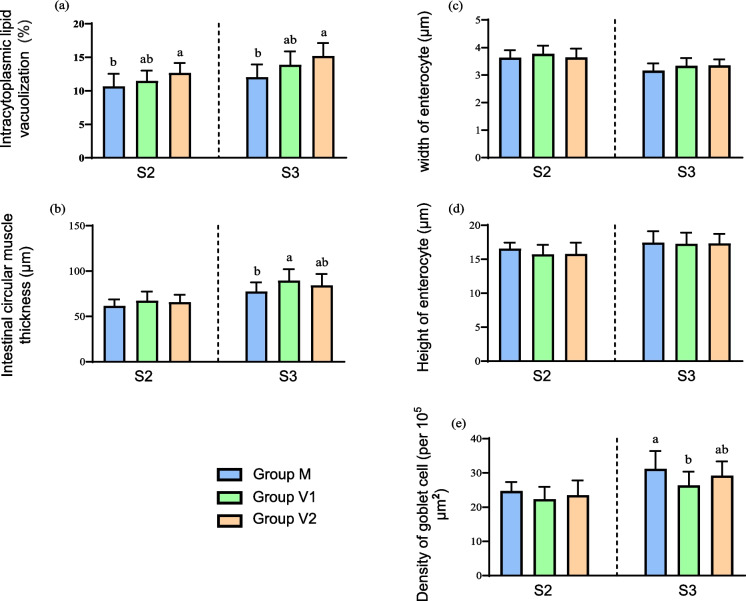
Histological analysis of liver (**a**) and anterior intestine (**b–e**) of Atlantic salmon at the end of the challenge phase. Columns represent mean values (*n* = 3) for the three dietary groups with standard deviation represented by vertical bars. Lowercase letters above bars denote significant differences (*p* < 0.05) among dietary groups for each histological parameter at the end of the intermediate (S2) or challenge (S3) phases

Histological analysis of the liver at the end of the challenge phase revealed that the length of the stimulus impacted hepatic intracytoplasmic lipid vacuolisation, with vacuolisation in the V2 group significantly higher than in the M group, with V1 demonstrating intermediary levels in both phases (Fig. 8a, Online Resource 2c-d). Fish in the V1 and V2 groups that had the V stimulus at first feeding had higher muscular thickness in anterior intestine than those in the M group (Fig. 8b), whereas the V stimulus at first feeding had no major impacts on enterocyte width or height at the end of either the intermediate or challenge phases (Fig. 8c, d). The density of goblet cells in the anterior intestine was significantly lower in the V1 group compared to the M group at the end of the challenge phase, while the V2 group showed intermediate values (Fig. 8e, Online Resource 2e-f).

## Discussion

Previously, we showed that feeding the vegetable diet for 3 weeks reduced the growth rate of salmon fry during the first feeding stimulus phase, resulting in smaller fry compared to fish fed a marine diet at first feeding, and the lower fish size persisted through the subsequent phase when all fish were fed the marine diet (Clarkson et al. 2017). Therefore, the present study aimed to investigate whether a stimulus phase of shorter duration could still induce beneficial programming effects without a negative effect on growth during this phase. However, salmon in the present study fed the vegetable diet for 1 or 2 weeks at first feeding also revealed lower body weights and SGR during the stimulus phase, indicating that short early nutritional interventions with vegetable (low marine ingredients) diets still impacted growth performance. Although growth was impacted, the results of the current study differed from those found in our earlier study (Clarkson et al. 2017), as growth performance in all treatment groups (V1, V2 and M) was comparable during both the intermediate and challenge phases. Indeed, although not statistically significant, growth performance data (weight gain and SGR) were numerically higher in V1 fish compared to M and V2 fish during both these phases while feed intakes were similar. The slightly better growth performance of V1 fish suggested that the 1-week stimulus might have had a potential positive effect on growth during the challenge phase. Early exposure to the vegetable-based diet may alter transcriptional or physiological characteristics and trigger olfaction imprinting to adapt the fish to this diet (Balasubramanian et al. 2016), leading to a better acceptance of the diet later in the growth cycle and, consequently, improve growth.

The lipid and fatty acid compositions of whole fish at the end of the stimulus phase indicated biochemical impacts of the early dietary intervention. While there were no differences in lipid levels between the three treatment groups, fatty acid compositions showed clear differences due to the duration of the stimulus phase. Therefore, the 1-week stimulus induced only minor changes in fatty acid composition of total lipid of whole fish, with the only differences being lower EPA and n-3 PUFA:n-6 PUFA ratio in V1 fish compared to M fish. However, with the 2-week stimulus, the differences in fatty acid compositions were considerable, with proportions of all n-3 PUFA reduced and all n-6 PUFA increased in the V2 fish compared to M fish. These changes were, of course, the likely response to the intake of the vegetable-based diet, but it was clear that, while they were occurring by 1 week, they took 2 weeks to develop fully.

Similar to the end of the stimulus phase, lipid contents after the challenge phase showed no differences in any tissue between the three treatment groups. However, histological examination of the liver revealed that the level of lipid vacuoles was higher in V2 fish that had the longer duration of stimulus. In liver, PPARα is a nuclear receptor regulating fatty acid oxidation and transport through target genes including CPT1 as the key rate-limiting enzyme of β-oxidation in mitochondria (Song et al. 2010). In the present study, both *pparα* and *cpt1* (and *aco*) showed the same pattern of lower expression in the liver of V2 fish than M fish at the end of the challenge phase, which was consistent with the 2-week stimulus affecting liver lipid metabolism via a mechanism involving *pparα* and *cpt1.* In contrast to *pparα*, the expression of *pparγ* was downregulated significantly in both V1 and V2 fish at the end of the challenge phase, and in V2 fish only at the end of the intermediate phase compared to M fish. Regulation of this nuclear receptor in liver can be influenced by gut microbiota driven by a change in diet (Murakami et al. 2016). In the present study, the stimulus V diet was found to have a sustained influence on the gut microbiota of V2 fish throughout the trial, especially at the end of the intermediate phase; although, unfortunately, this was not studied in V1 fish (Tawfik et al. 2024). Higher hepatic intracytoplasmic lipid vacuolisation at the end of the challenge could be related to the extended length of time that *pparγ* was downregulated. However, while the lower expression of these genes in V2 fish was associated with higher hepatic intracytoplasmic lipid vacuolisation, liver lipid content was not statistically different among treatment groups. It is possible that, although gene expression levels were lower in V2 fish, the degree of downregulation was insufficient to translate into detectable biochemical changes within the relatively short duration of the challenge phase and, therefore, a longer challenge period may be required in future studies.

Of course, the nutritional factors/nutrients that the present study focused on were n-3 LC-PUFA and so fatty acid composition, specifically EPA and DHA content, was a key indicator of the success of nutritional programming and the effect of duration of stimulus. The aim of nutritional programming in this respect being to mitigate the negative effects of reduced n-3 LC-PUFA in low-marine diets. As the fatty acid composition of PL is more conserved, reflecting the roles of PL in physiological and metabolic functions, compared to the composition of TL that more reflects the diet (Bell et al. 1996; Betancor et al. 2014), we hypothesised that the fatty acid compositions of PL and TL would reflect this functional difference and respond differently to nutritional programming. However, the fatty acid compositions at the end of the challenge phase showed no significant differences between the V1, V2 and M treatment groups in either TL or PL in any of the analysed tissues. Therefore, the fatty acid composition data alone could neither indicate the ideal stimulus duration, nor if the fish from V1 and V2 were programmed.

Nevertheless, in contrast, it was notable that many significant differences were detected in gene expression in liver. Comparison of liver fatty acid composition between the intermediate and challenge phases showed a marked increase in ARA content after challenge than before (5% *vs*. 2%) while dietary ARA during the challenge phase was only around 0.1%. So, the accumulation of ARA was likely due largely to endogenous biosynthesis from the abundant LA in the V diet, which was supported by the significantly increased level of 20:3n-6 intermediate, indicating activation of the LC-PUFA biosynthesis pathway as reported previously (Izquierdo et al. 2008; Tocher et al. 2002, 2003). Furthermore, diets low in EPA and DHA have been shown previously to induce greater deposition of ARA and 20:3n-6 in PL of liver and other tissues in Atlantic salmon (Bou et al. 2017). However, as indicated above, there were no differences in ARA level after challenge between treatments despite expression levels of *fads* and *elovls* being higher in V1 compared to M fish, with V2 similar to M fish. The result for V1 fish was consistent with the study of Turkmen et al. (2020) who demonstrated increased expression of ∆6 *fads* in offspring when broodstock were programmed by a high ALA and LA diet. It is not clear why the expression of the *fads* and *elovl* genes was reduced in V2 fish with longer exposure to the V diet at first feeding than the V1 fish. Expression of the LC-PUFA genes in liver reported here was different from expression levels in pyloric caeca of fish in the same trial reported previously, which showed a trend for lower expression in V1 compared to V2 fish, although all V fish showed lower expression than M fish (McMillan et al. 2024). This suggested that dietary stimuli may have differential impacts in different tissues depending upon functional roles. The expression levels of *fads* and *elovl* in liver were in line with expression levels of *lxr* and *srebp*, which may be expected because *srebp* is the target gene of *lxr*. Both genes are sensors of fatty acid metabolism balance (Horton et al. 2003; Tobin et al. 2002) and are involved in PUFA desaturation and elongation through transcriptional regulation of ∆6, ∆5 *fads* (Matsuzaka et al. 2002; Zheng et al. 2009) and *elovl5* (Moon et al. 2009; Qin et al. 2009). In this respect, how nutritional programming could affect LC-PUFA biosynthesis is interesting, with the precise mechanism potentially involving modification of either *fads* and *elovl* directly, or via their regulatory upstream genes, or both. Previously, epigenetic changes were reported in the *fads2* gene with methylation in the promoter in offspring being correlated with the parental diet in gilthead sea bream (Turkmen et al*.* 2019).

Histological analysis of intestine, as the key organ of nutrient digestion and absorption, at the end of the challenge phase also displayed differences due to dietary treatment during the stimulus phase that may suggest nutritional programming. Salmon subjected to the V diet stimulus, especially for 1 week (V1 fish), showed thicker intestinal muscular layer than M fish at the end of the challenge phase. Vegetable-based diets can potentially take effects; previous research demonstrated that replacement of 50–70% of FM by soybean meal decreased the muscular layer thickness in the intestine of Japanese sea bass (*Lateolabrax japonicus*) (Zhang et al. 2018). Another report presented that 80% FO replaced by VO did not impact the thickness of mid-intestinal circular muscle, but the thickness was decreased in the distal intestine (Moldal et al. 2014). Therefore, in the present study, application of the low marine (V) diet in the challenge phase did not negatively affect intestinal muscle thickness, but actually increased it in V fish compared to M fish. This difference can only be attributed to the one difference between the different fish groups, namely the early application of the low-marine V diet to V1 and V2 fish in the stimulus phase. Thus, this appears to support the theory of nutritional programming to prepare the animals for future dietary changes. Similarly, the density of goblet cells was lower in the intestine of V1 fish compared to M fish. Goblet cells are specialised columnar epithelial cells that, while being intimately involved in digestion, also provide innate protection against chemical substances through the secretion of mucus as a barrier layer, and cytokines that activate intestinal immunity (Kim and Ho 2010; Deplancke and Gaskins 2001). Previous studies showed that plant-based diets reduced goblet cell numbers and mucus production in Atlantic salmon, rainbow trout and Nile tilapia (*Oreochromis niloticus*) (Navarrete et al. 2013; Obirikorang et al. 2020; Venold et al. 2012). Therefore, goblet density is associated positively with intestinal health, immune response, and nutrient digestion, and therefore, the lower density of goblet cells in V1 fish compared to M fish in the present study suggested weaker mucus protection in the intestine and potentially increased susceptibility to bacterial disease. This implied that the intestinal impacts of the V diet at stimulus may be a double-edged sword, positively improving digestion, but negatively affecting intestinal immune status. However, in addition to goblet cell number, cell size could provide additional insights in relation to mucus production and intestinal function and, therefore, should also be considered in future studies.

While the above discussion has highlighted that the different diets (V and M) applied briefly at first feeding resulted in differences in intestinal structure and liver gene expression when fish were subsequently challenged with the V diet, it is acknowledged that the primary aim of the study, to determine the impact of the duration of the first feeding stimulus, was difficult to confirm. At first feeding, 1 week of the V diet had only minor effects on the fatty acid composition of the fry, while 2 weeks had a major impact, which was highlighted and emphasised clearly by PCA. Assuming that the physiological consequences of the dietary intervention would have to be based on impacts on fatty acid compositions, this would suggest that 1-week (V1) might not be sufficient to induce nutritional programming, and that at least 2 weeks would be required. However, the impacts of the dietary stimulus on the intestine do not fully support this, as intestinal histology was also more impacted in V1 fish than V2 fish, albeit the effects were both potentially positive and negative. Liver gene expression also tended to suggest a better outcome in V1 fish, although the effects were similarly complicated. Overall, although the expression levels of many lipid-related genes in liver were consistently lower in V2 fish compared to V1 fish in the challenge phase, and levels in V1 fish were generally similar to those in M fish, this was not the case for the LC-PUFA biosynthesis genes, where expression was highest in V1 fish. However, there were no differences in tissue fatty acid compositions between V1 or V2 or M fish at the end of the challenge phase and, therefore, no conclusive data to support either duration. However, although differences in EPA and DHA retention between M and V fish were reported in both our previous study (Clarkson et al. 2017) and the present study (McMillan et al. 2024), this was not reflected at the phenotypic level in the fish or tissue fatty acid compositions in either study. Therefore, the fact that many signs of nutritional programming over two studies have not been reflected in fatty acid compositions may suggest that the challenge phase (6 weeks) may not be of sufficient duration. Typically, fish feeding trials would be assessed after 8–12 weeks, albeit this varies with the size of the fish (Turchini and Hardy 2024), but a threefold increase in growth is recommended (NRC 2011). In the present study, while growth over the entire trial far exceeded this, growth over the challenge phase was only twofold (McMillan et al. 2024). Furthermore, it is well-established that changes in the fatty acid profile of fish following changes in diet fatty acid composition essentially follow a dilution model (Jobling 2003, 2004; Robin et al. 2003). These models have been tested in fish including Atlantic salmon with switches from FO to VO diets, and show that dilution is the predominant driver of the resultant fish fatty acid profile over long periods of up to 14 weeks depending upon the size of the fish (Jobling 2003, 2004). Thus, in the present study, low EPA and DHA and high ALA and LA dominate the dietary fatty acid changes when switched from the marine M diet to the vegetable V diet, and dilution of EPA and DHA will be the dominant driver of their tissue levels in fish, masking the genetically driven molecular/biochemical responses.

In conclusion, the present study sought to provide insight into whether the duration of an early dietary stimulus significantly impacted nutritional programming. Despite few phenotypic data to support programming, liver gene expression and liver and intestinal histology showed that diet composition at first feeding and the duration of the stimulus had measurable impacts. On balance, the data may suggest that the V1 salmon were better adapted to plant-based diets than V2 fish as, after challenge, genes encoding LC-PUFA biosynthetic enzymes were upregulated in V1 fish compared to V2 fish. Furthermore, changes in intestine suggested fish that experienced the 1-week stimulus may have better potential in nutrient absorption, although they may also have weakened immune status. Considering the lack of phenotypic effects observed after the 6-week challenge phase, it is suggested that future studies in salmon investigating nutritional programming for fatty acid utilisation at first feeding should include a longer challenge phase to aid assessment. In addition, the molecular mechanisms of nutritional programming, including the epigenome of regulatory and target genes, require further in-depth study.

## Supplementary Information

Below is the link to the electronic supplementary material.

ESM 1 PDF (198 KB)

ESM 2 PDF (1.27 MB)

## Data Availability

The datasets are available from the author Xu Gong on reasonable request.

## Abbreviations

ALA: α-Linolenic acid (18:3n-3)
ANF: Antinutritional factor
ARA: Arachidonic acid (20:4n-6)
DD: Degree-days
DHA: Docosahexaenoic acid (22:6n − 3)
EFA: Essential fatty acid
EPA: Eicosapentaenoic acid (20:5n − 3)
∆6 FAD: Delta-6-desaturase
FAME: Fatty acid methyl ester
FM: Fishmeal
FO: Fish oil
GOI: Genes of interest
LA: Linoleic acid (18:2n-6)
LC-PUFA: Long-chain polyunsaturated fatty acids
M: Marine diet
MUFA: Monounsaturated fatty acid
PL: Polar lipid
SGR: Specific growth rate
TAG: Triacylglycerol
TL: Total lipid
V1: Plant-based diet for 1 week
V2: Plant-based diet for 2 weeks

## Declarations

### Disclaimer

The authors confirm that the manuscript has not been published before and has been read and approved.
