# Supplementary material for: Effects of duration of a plant-based diet stimulus at first feeding on nutritional programming in Atlantic salmon (Salmo salar)
Source: Fish Physiol Biochem. 2026 Feb 17;52(2):33. doi: 10.1007/s10695-026-01639-7 (PMC12913327; doi:10.1007/s10695-026-01639-7)
Supplement: Supplementary file 1 — PDF (198 KB) [file 10695_2026_1639_MOESM1_ESM.pdf]

Title: Effects of duration of a plant-based diet stimulus at first feeding on nutritional programming in Atlantic salmon (*Salmo salar*)

Journal: Fish Physiology and Biochemistry

Author names: Xu Gong[1], Matthew Sprague[1], Stuart McMillan[1], Pedro Gómez Requeni[2], Fernando Norambuena[3], Sam A M Martin[4], Douglas R Tocher[1,5], Mónica B Betancor[1*]

Affiliation:

[1] Institute of Aquaculture, School of Natural Sciences, University of Stirling, Stirling FK9 4LA, UK

[2] BioMar A/S, Mylius Erichsensvej 35, Brande 7330, Denmark

[3] BioMar AS, Havnegata 9, Pirsenteret 3, Trondheim 7010, Norway

[4] School of Biological Sciences, University of Aberdeen, Aberdeen, AB24 2TZ, UK

[5] Guangdong Provincial Key Laboratory of Marine Biotechnology, Shantou University, Shantou 515063, Guangdong, China

*Corresponding author Tel.: + 44 1786 467892

E-mail: m.b.betancor@stir.ac.uk

Online Resource 1 Sequences of gene primers used for qPCR including amplicon sizes and annealing temperatures (Tm)

Category	Gene	Forward sequence (5'→3')	Reverse sequence (3'→5')	Amplicon	Tm (ºC)	Accession no.
Fatty acid biosynthesis related	*elovl2*	GGTGCTGTGGTGGTACTACT	ACTGTTAAGAGTCGGCCCAA	190	59	NM_001136553.1[a]
	elovl5a	TGTTGCTTCATTGAATGGCCA	TCCCATCTCTCCTAGCGACA	150	59	GU238431.1[a]
	elovl5b	CTGTGCAGTCATTTGGCCAT	GGTGTCACCCCATTTGCATG	192	59	NM_001136552.1[a]
	fads2d5	GCCACTGGTTTGTATGGGTG	TTGAGGTGTCCACTGAACCA	148	59	NM_001123542.2[a]
	fads2d6	TCCTCTGGTGCGTACTTTGT	AAATCCCGTCCAGAGTCAGG	163	59	NM_001123575.2[a]
Transcription factors	*lxr*	GCCGCCGCTATCTGAAATCTG	CAATCCGGCAACCAATCTGTAGG	210	58	FJ470290[a]
	pparα	TCCTGGTGGCCTACGGATC	CGTTGAATTTCATGGCGAACT	111	60	DQ294237[a]
	pparγ	CATTGTCAGCCTGTCCAGAC	TTGCAGCCCTCACAGACATG	144	60	AJ416951[a]
	srebp1	GCCATGCGCAGGTTGTTTCTTCA	TCTGGCCAGGACGCATCTCACACT	151	63	TC148424[b]
	srebp2	TCGCGGCCTCCTGATGATT	AGGGCTAGGTGACTGTTCTGG	147	63	TC166313[b]
Lipid catabolism related	*aco*	AAAGCCTTCACCACATGGAC	TAGGACACGATGCCACTCAG	230	60	TC49531[b]
	cptI	CCTGTACCGTGGAGACCTGT	CAGCACCTCTTTGAGGAAGG	212	60	AM230810[a]
	fas	ACCGCCAAGCTCAGTGTGC	CAGGCCCCAAAGGAGTAGC	212	60	CK876943[a]
	hmgcr	CCTTCAGCCATGAACTGGAT	TCCTGTCCACAGGCAATGTA	224	60	DW561983[a]
Housekeeping genes	*ef1a*	CTGCCCCTCCAGGACGTTTACAA	CACCGGGCATAGCCGATTCC	175	60	AF321836[a]
	hprt	GATGATGAGCAGGGATATGAC	GCAGAGAGCCACGATATGG	165	60	BT125296.1
	rpl2	CTGCCCCTCCAGGACGTTTACAA	TGTTCACAGCTCGTTTACCG	112	60	XM_014137227.1[a]

Genes of Interest: *aco*, acyl-CoA oxidase; *cptI*, carnitine palmitoyltransferase I; *elovl2*, fatty acyl elongase 2; *elovl5a*, fatty acyl elongase 5 isoform a; *elovl5b*, fatty acyl elongase isoform b; *fas*, fatty acid synthase; *fads2d5*, delta-5 fatty acyl desaturase; *fads2d6*, delta-6 fatty acyl desaturase; *hmgcr*, 3-hydroxy-3-methyl-glutaryl-CoA reductase; *lxr*, liver X receptor; *pparα*, peroxisome proliferator-activated receptor alpha; *pparγ*, peroxisome proliferator-activated receptor gamma; *srebp1*, sterol regulatory element binding protein 1; *srebp2*, sterol regulatory element binding protein 2.

Housekeeping: *ef1α*, elongation factor 1 alpha; *hprt*, hypoxanthine-guanine phosphoribosyltransferase; *rpl2*, ribosomal protein L2; They were considered as the most stable according to the geNorm.

[a]GenBank (http://www.ncbi.nlm.nih.gov/); [b]Atlantic salmon Gene Index (http://compbio.dfci.harvard.edu/tgi/)
