# Supplementary material for: Effects of duration of a plant-based diet stimulus at first feeding on nutritional programming in Atlantic salmon (Salmo salar)
Source: Fish Physiol Biochem. 2026 Feb 17;52(2):33. doi: 10.1007/s10695-026-01639-7 (PMC12913327; doi:10.1007/s10695-026-01639-7)

Title: Effects of duration of a plant-based diet stimulus at first feeding on nutritional programming in Atlantic salmon (*Salmo salar*)

Journal: Fish Physiology and Biochemistry

Author names: Xu Gong[1], Matthew Sprague[1], Stuart McMillan[1], Pedro Gómez Requeni[2], Fernando Norambuena[3], Sam A M Martin[4], Douglas R Tocher[1,5], Mónica B Betancor[1*]

Affiliation:

[1] Institute of Aquaculture, School of Natural Sciences, University of Stirling, Stirling FK9 4LA, UK

[2] BioMar A/S, Mylius Erichsensvej 35, Brande 7330, Denmark

[3] BioMar AS, Havnegata 9, Pirsenteret 3, Trondheim 7010, Norway

[4] School of Biological Sciences, University of Aberdeen, Aberdeen, AB24 2TZ, UK

[5] Guangdong Provincial Key Laboratory of Marine Biotechnology, Shantou University, Shantou 515063, Guangdong, China

*Corresponding author Tel.: + 44 1786 467892

E-mail: m.b.betancor@stir.ac.uk

Online Resource 2 The illustration of the measurement detail (a, b), and the vacuolization in Atlantic salmon liver of M (c) and V2 (d) along with the goblet cells in anterior intestine of M (e) and V1 (f) fish at the end of the challenge phase, respectively

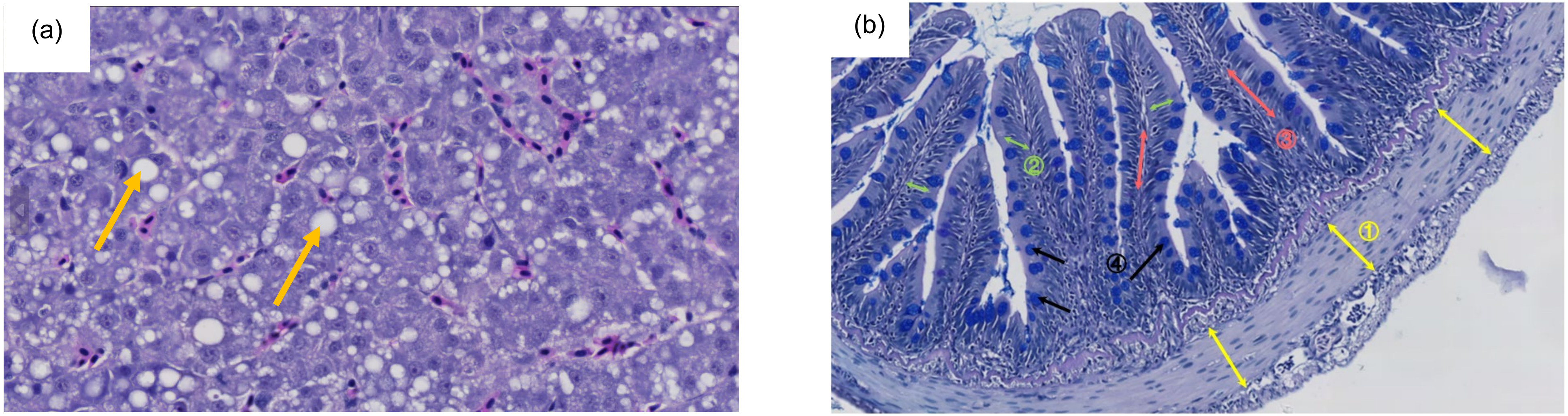

* The intracytoplasmic lipid vacuolisation (orange arrows)

① The measurements of intestinal circular muscle thickness (yellow arrows)
② The measurements of enterocyte height (green arrows)
③ Areas of counting enterocyte amounts for the width measurements (red arrows)
④ Goblet cell (black arrows)

Online Resource 2 Cont

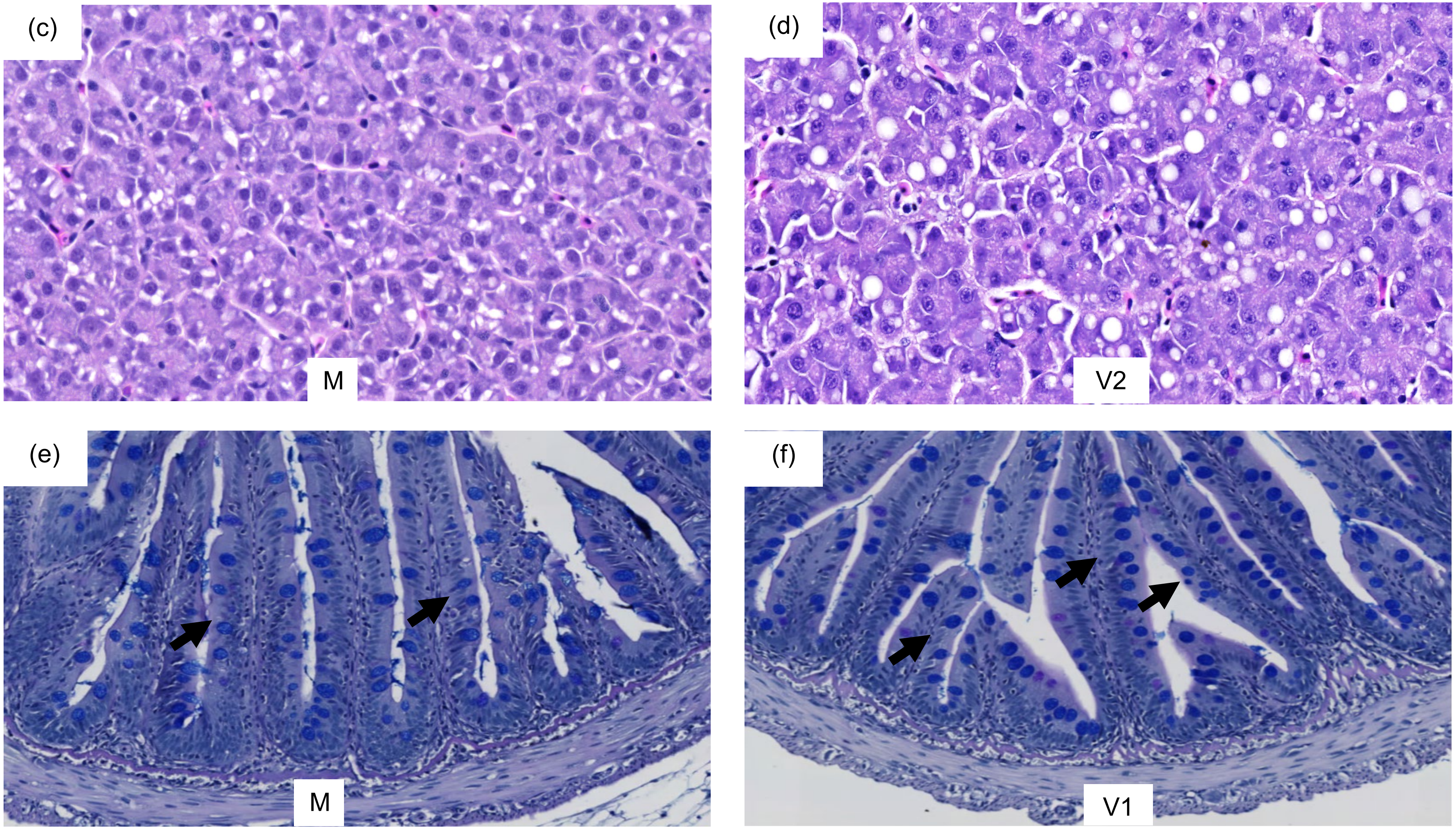

Supplement: Supplementary file 2 — PDF (1.27 MB) [file 10695_2026_1639_MOESM2_ESM.pdf]
